# Straight and rigid flagellar hook made by insertion of the FlgG specific sequence into FlgE

**DOI:** 10.1038/srep46723

**Published:** 2017-04-21

**Authors:** Koichi D. Hiraoka, Yusuke V. Morimoto, Yumi Inoue, Takashi Fujii, Tomoko Miyata, Fumiaki Makino, Tohru Minamino, Keiichi Namba

**Affiliations:** 1 Graduate School of Frontier Biosciences, Osaka University, 1-3 Yamadaoka, Suita, Osaka 565-0871, Japan; 2 Riken Quantitative Biology Center, 1-3 Yamadoaka, Suita, Osaka 565-0871, Japan

## Abstract

The bacterial flagellar hook connects the helical flagellar filament to the rotary motor at its base. Bending flexibility of the hook allows the helical filaments to form a bundle behind the cell body to produce thrust for bacterial motility. The hook protein FlgE shows considerable sequence and structural similarities to the distal rod protein FlgG; however, the hook is supercoiled and flexible as a universal joint whereas the rod is straight and rigid as a drive shaft. A short FlgG specific sequence (GSS) has been postulated to confer the rigidity on the FlgG rod, and insertion of GSS at the position between Phe-42 and Ala-43 of FlgE actually made the hook straight. However, it remains unclear whether inserted GSS confers the rigidity as well. Here, we provide evidence that insertion of GSS makes the hook much more rigid. The GSS insertion inhibited flagellar bundle formation behind the cell body, thereby reducing motility. This indicates that the GSS insertion markedly reduced the bending flexibility of the hook. Therefore, we propose that the inserted GSS makes axial packing interactions of FlgE subunits much tighter in the hook to suppress axial compression and extension of the protofilaments required for bending flexibility.

The bacterial flagellum is a supramolecular motility machine consisting of the basal body, the hook and the filament. The basal body is embedded in the cell surface and functions as a reversible rotary motor powered by proton or monovalent-cation motive force across the cytoplasmic membrane. In Gram-negative bacteria, such as *Salmonella enterica*, the basal body consists of the MS-C ring complex as a reversible rotor, the LP ring complex as a molecular bushing, and the rod as a drive shaft. The hook and filament extend out into the cell exterior, and the filament functions as a helical propeller. The hook connects the basal body with the filament and acts as a universal joint to smoothly transmit motor rotation to the filament that changes its orientation dynamically during swimming and tumbling of the cell^1^,^2^,^3^. So the hook is curved and flexible in bending whereas the rod is straight and rigid as a drive shaft.

The rod is composed of three proximal rod proteins FlgB, FlgC, FlgF and the distal rod protein FlgG^4^. FliE forms the MS ring/rod junction zone at the periplasmic surface of the MS ring made of a transmembrane protein, FliF^5^. Rod assembly begins with FlgB on the FliE junction zone, followed by FlgC and FlgF in this order to form the proximal rod, and finally FlgG forms the distal rod^6^. FlgJ not only acts as the rod cap to promote rod assembly^7^ but also hydrolyzes the peptidoglycan layer for the rod to penetrate through the peptidoglycan layer^8^. The hook protein FlgE assembles at the distal end of the rod and the growing end of the hook with the help of the hook cap made of FlgD to form a 55 nm tubular structure of the hook^9^,^10^. Although the rod and hook have distinct mechanical properties, namely the rod being straight and rigid whereas the hook being supercoiled and flexible in bending, the hook is directly connected to the distal end of the FlgG rod^11^.

The length of *Salmonella* hook is controlled to be around 55 nm by a physical interaction between the ruler protein FliK and the export switch protein FlhB^12^,^13^. FliK is secreted via the flagellar type III export apparatus during hook assembly^14^, and its N-terminal portion binds to the hook cap with high affinity and the hook with low affinity in its unfolded form inside the central channel of the hook to measure the hook length^15^,^16^,^17^. When the hook has reached its mature length of 55 nm, temporal association of the N-terminal domain of FliK with the FlgD cap and the inner surface of the hook allows the C-terminal domain of FliK to interact with FlhB in the export gate located at the center of the MS ring, causing FlhB to switch the export substrate specificity from rod/hook type to filament type, thereby terminating the export of FlgE and initiating the export of proteins responsible for filament formation^18^,^19^,^20^.

FlgE is composed of four domains, D0, Dc, D1 and D2, arranged from the inner to the outer part of the hook structure, which is composed of 11 protofilaments^21^,^22^,^23^. The atomic model of the straight hook shows that the axial packing of the D1 and D2 domains in the outer part of the hook is relatively loose, which is primarily responsible for the bending flexibility of the hook structure^21^,^23^. The N- and C-terminal α-helices form a coiled coil in the inner core domain D0, and it is the extensive interactions between the terminal coiled coils of subunits in the inner part of the hook that are responsible for structural and mechanical stabilization of the hook structure^23^. It has been shown that the hook undergoes polymorphic transformations of its supercoiled form in response to changes in the salt concentration, pH and temperature of the solution^24^. The most plausible mechanism of hook supercoiling that has been suggested by the atomic model of a curved supercoiled hook is that the close axial packing of the D2 domains on the inner side of the maximally curved hook, which is made possible by its bending flexibility, is responsible for the supercoiling^21^. Thus, the curvature and twist of each supercoil presumably depends on the direction of intermolecular D2-D2 interactions in the protofilaments, suggesting that hook supercoiling could not occur if the bending flexibility is reduced or domain D2 is removed.

The amino acid sequence of FlgG shows a high degree of identity (39%) with parts of the sequence of FlgE covering its domains D0, Dc and D1^25^,^26^. Consistently, high-resolution structural analysis of the FlgG polyrod by electron cryomicroscopy and helical image analysis has shown that the rod has the same helical symmetry and repeat distance as those of the hook and that domains D0 and D1 of FlgG adopt tertiary folds nearly identical to domains D0 and D1 of FlgE, respectively^26^. However, one major structural difference between the rod and hook is the orientation of their D1 domains relative to the tubular axis, resulting in their tight and loose axial packing in the rod and hook, respectively, and this difference is likely to be responsible for the bending rigidity of the rod and flexibility of the hook^26^. The N-terminal part of FlgG corresponding to domain Dc of FlgE, formed mainly by residues 25–70, has a FlgG specific sequence (GSS) consisting of 18 residues (YQTIRQPGAQSSEQTTLP) not present in FlgE (Fig. 1a)^25^. Since insertion of this GSS between Phe-42 and Ala-43 of FlgE makes the hook straight, the GSS appears to be responsible for the rigidity of the FlgG rod by making the orientation of the D1 domains in such a way for them to form much tighter axial packing interactions than those in the hook structure^26^. Systematic deletion analyses of the terminal regions of FlgE have shown that structural perturbations of domain Dc affect not only hook assembly but also its supercoiling^27^, suggesting that domain Dc plays an important role in determining the orientation of domain D1, as suggested by structural comparison between the FlgG rod and the hook^26^. However, it remains unclear whether the GSS insertion into FlgE actually makes the hook more rigid by suppressing the compression and extension of each protofilament of the hook structure.

In the present study, to understand the role of the Dc domain of FlgE in the universal joint mechanism in more detail, we characterized a *Salmonella flgE* mutant (*flgE*_+*GSS*_), which has the extra 18 residues insertion between Phe-42 and Ala-43 of FlgE. We show that the GSS insertion makes the hook straight and considerably reduces the probability to form a flagellar bundle behind the cell body, thereby impairing the swimming motility. We suggest that the GSS insertion makes the hook much less flexible in bending.

## Results

### Characterization of a *flgE*_+*GSS*_ strain

**Figure 1:**
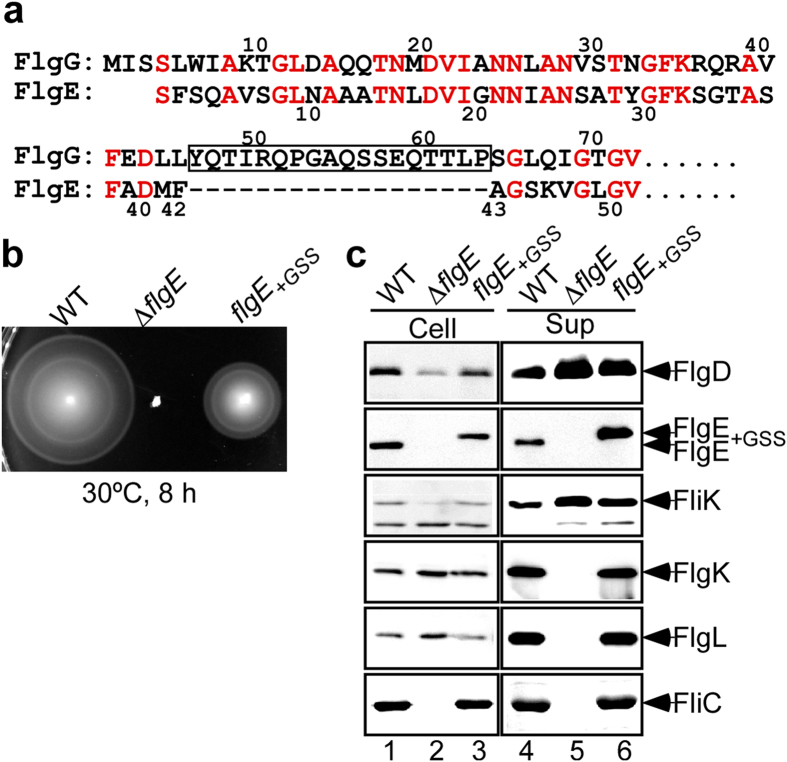
Characterization of the *flgE*_+*GSS*_ mutant. (**a**) Sequence alignment of the N-terminal regions of FlgE and FlgG. The FlgG-rod specific sequence is indicated by a box. (**b**) Motility of SJW1103 (WT), NME001 (∆*flgE*) and MME1001 (*flgE*_+*GSS*_). Soft tryptone agar plates were incubated at 30 °C for 8 h. (**c**) Secretion properties of the *flgE*_+*GSS*_ mutant. Immunoblotting, with polyclonal anti-FlgD, anti-FlgE, anti-FliK, anti-FlgK, anti-FlgL or anti-FliC antibody, of the whole cellular (Cell) and culture supernatant (Sup) fractions prepared from the above strains. Each cropped blot was shown by a box.

To examine whether the GSS consisting of 18 residues, YQTIRQPGAQSSEQTTLP (Fig. 1a), plays an important role in conferring rigidity on the FlgG rod, we first analyzed motility of the *flgE*_+*GSS*_ mutant, which has the GSS insertion between Phe-42 and Ala-43 of FlgE in soft agar to see the function of this mutant hook (Fig. 1b). The *flgE*_+*GSS*_ mutant was motile although not at the level of the wild-type strain. This indicates that the GSS insertion somehow perturbs the functional property of the hook as a universal joint.

It has been shown that residues 30–49 of FlgE are required for rapid and efficient FlgE export^27^. We tested whether the GSS insertion into the Dc domain of FlgE affects the export of FlgE. When the hook has reached its mature length of ca. 55 nm, the flagellar type III export apparatus switches export specificity from hook-type (FlgD, FlgE and FliK) to filament-type substrates (FlgM, FlgK, FlgL, FliC, FliD), thereby terminating hook polymerization and starting filament formation^12^,^13^. Because assembly-deficient *flgE* mutants do not produce the hook structure and hence cannot change substrate specificity of the export apparatus, they secrete much higher amounts of the hook-type proteins into the culture media than wild-type cells^28^,^29^. Therefore, we analyzed the levels of the hook-type proteins secreted by the *flgE*_+*GSS*_ strain (Fig. 1c). Immunoblotting with polyclonal anti-FlgD (first row), anti-FlgE (second row) and anti-FliK (third row) antibodies revealed that the secretion levels of FlgD, FlgE and FliK by the *flgE*_+*GSS*_ cells (lane 6) were much higher than the wild-type levels (lane 4) and slightly less than those by the ∆*flgE* mutant cells (lane 5). Since the increased levels of FlgE secretion by weakly motile *flgE* mutants reflect a decrease in the polymerization ability of mutant FlgE proteins into hooks^15^, we suggest that the GSS insertion reduces the rate of hook polymerization but not the export rate of FlgE at all.

Filament-type proteins are exported only after the completion of hook assembly^12^,^13^. Therefore, we analyzed proteins secreted by the *flgE*_+*GSS*_ cells by immunoblotting with polyclonal anti-FlgK (forth row), anti-FlgL (fifth row), or anti-FliC antibody (sixth row). FlgK, FlgL and FliC were detected almost at wild-type levels in the culture supernatants of the *flgE*_+*GSS*_ strain (lane 6) but not in the ∆*flgE* mutant (lane 5). Therefore, we conclude that the *flgE*_+*GSS*_ cells form hooks and subsequently switch the substrate specificity of the export apparatus upon completion of hook assembly.

**Figure 2:**
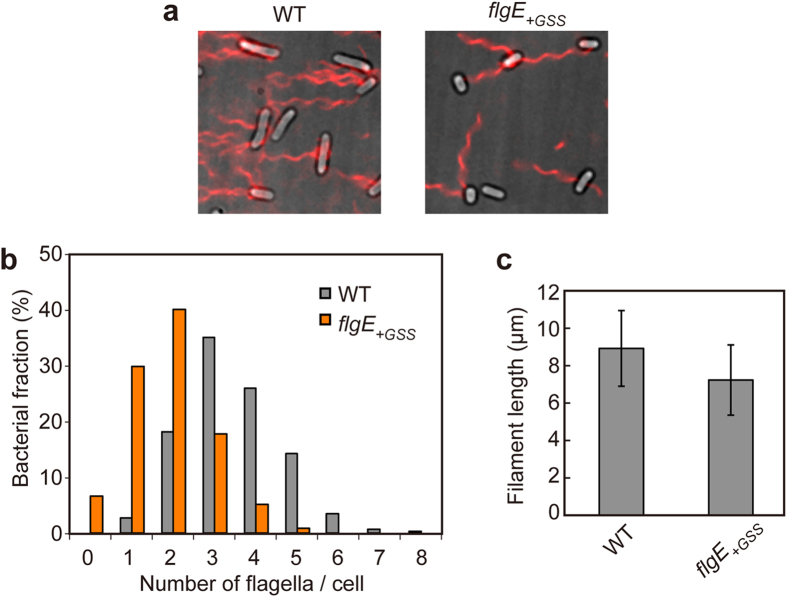
Effect of the GSS insertion on the number and length of the flagellar filaments. (**a**) Fluorescence images of flagellar filaments produced by SJW1103 (WT) and MME1001(*flgE*_+*GSS*_). *Salmonella* cells were grown exponentially and then flagellar filaments were labeled with polyclonal anti-FliC antibody and anti-rabbit IgG conjugated with Alexa Fluor 594. The fluorescence images of the filaments labeled with Alexa Fluor 594 (red) were merged with the bright field images of the cell bodies. (**b**) Distribution of the number of the flagellar filaments in SJW1103 (WT) and MME1001(*flgE*_+*GSS*_). More than 260 cells for each strain were counted. (**c**) Measurements of the length of the flagellar filaments produced by each strain. Filament length is the average of 100 cells, and vertical lines are standard deviations.

We then quantitatively analyzed the number of the flagellar filaments produced by the *flgE*_+*GSS*_ cells (Fig. 2a). The number of filaments produced by wild-type cells ranged from 1 to 8 with an average of 3.5 ± 1.2 (Fig. 2b). More than 90% of the *flgE*_+*GSS*_ cells produced filaments with the number ranged from 1 to 5 with an average of 1.9 ± 1.0 (Fig. 2b). The average filament length was slightly shorter than the wild-type length (Fig. 2c), suggesting that the GSS insertion affect the timing of filament growth initiation due to the reduced rate of hook assembly. Therefore, we conclude that the *flgE*_+*GSS*_ strain is a slow hook assembly mutant.

### Effects of the GSS insertion on the hook supercoiling

**Figure 3:**
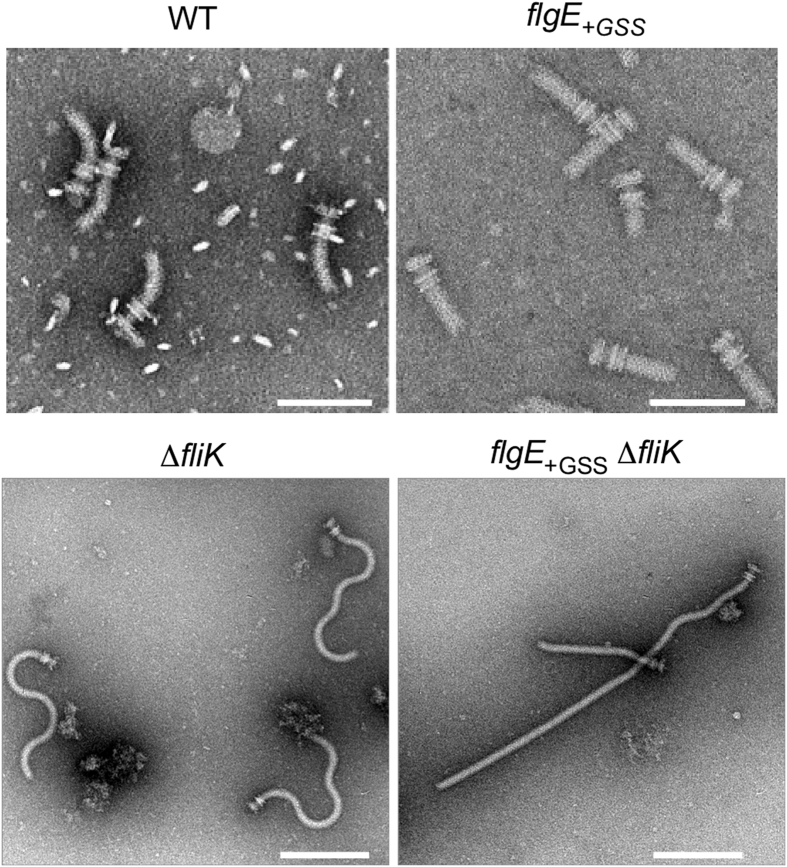
Effect of the GSS insertion on hook assembly and morphology. Electron micrographs of the hook-basal bodies produced by wild-type (WT) and the *flgE*_+*GSS*_ mutant (scale bar, 100 nm) and the polyhook-basal bodies produced by the ∆*fliK* mutant and the *flgE*_+*GSS*_ ∆*fliK* double mutant (scale bar, 200 nm).

The polyhook is an abnormally long hook produced by mutant strains lacking the ability to control hook length^30^ and shows polymorphic transformations between its supercoiled forms^24^. It has been shown that the Dc domain of FlgE affects the hook supercoiling^27^. To investigate how the GSS insertion affects the morphology of the hook and its mechanical property, we isolated the flagellar hook-basal bodies from the wild-type and *flgE*_+*GSS*_ mutant cells (Fig. 3). Since mutations in FliK produces polyhooks^30^,^31^, we also isolated polyhook-basal bodies from the ∆*fliK*::*tetRA* and *flgE*_+*GSS*_ ∆*fliK*::*tetRA* double mutant cells to evaluate the bending flexibility of the hook (Fig. 3). The hooks and polyhooks produced by the wild-type and ∆*fliK*::*tetRA* mutant cells, respectively, adopted highly curved and supercoiled conformations, respectively. In agreement with a previous report^26^, the *flgE*_+*GSS*_ and *flgE*_+*GSS*_ ∆*fliK*::*tetRA* mutant cells produced straight hooks and straight polyhooks, respectively. These results indicate that the GSS insertion into FlgE makes the hook straight and much less flexible in its bending to suppress supercoiling.

### Effects of the GSS insertion on free swimming and flagellar bundle formation

**Figure 4:**
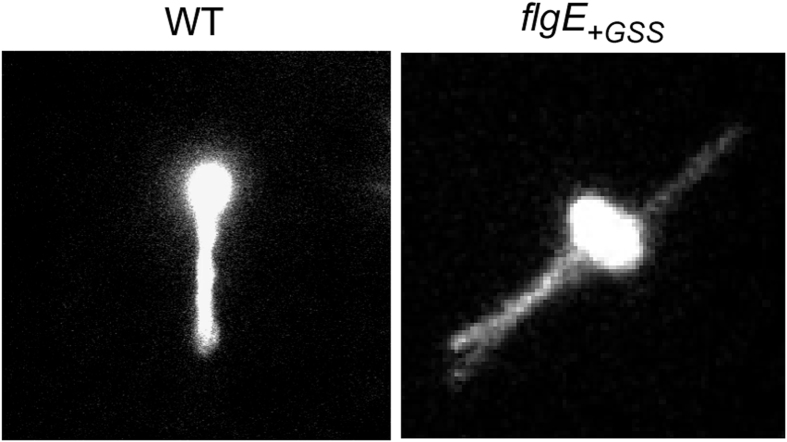
Effect of the GSS insertion on flagellar bundle formation. Fluorescence images of swimming cells: SJW1103 (WT) and MME1001(*flgE*_+*GSS*_). The cells were grown in LB until their growth had reached the late exponential phase or the early stationary phase. The cell bodies and flagellar filaments were stained with a Cy3 mono-reactive dye. (See Supplementary movies S1 and S2).

The bending flexibility of the hook structure allows the filaments to form a bundle behind the cell body to produce thrust^32^. To further test how much the bending flexibility of the hook is affected by the GSS insertion, we stained the cell body and flagellar filaments with a Cy3 mono-reactive dye and observed free-swimming behavior of the wild-type and *flgE*_+*GSS*_ mutant cells under a fluorescence microscope (Fig. 4 and Supplementary movies S1 and S2). Since the hook polymerization rate of the *flgE*_+*GSS*_ mutant was slower than the wild-type and hence the timing of filament growth initiation was simply delayed (Figs 1 and 2), both the wild-type and *flgE*_+*GSS*_ mutant cells were grown until they had entered the late exponential growth phase or the early stationary phase. The wild-type cells efficiently formed a flagellar bundle behind the cell body and swam smoothly. In contrast, the *flgE*_+*GSS*_ cells were unable to form the flagellar bundle properly, with each filament extending in different directions, and were not able to swim smoothly. Therefore, we conclude that the GSS insertion significantly reduces the bending flexibility of the hook.

## Discussion

The tertiary folds of domains D0, Dc and D1 of FlgG are nearly identical to domains D0, Dc and D1 of FlgE^25^,^26^, and the FlgG rod has the same helical symmetry and repeat distance as those of the hook^26^. The FlgG rod acts as a drive shaft whereas the hook acts as a universal joint to transmit motor torque to the filament in its dynamically changing orientations during cell swimming and tumbling. The D1 domains of FlgG show tight axial packing interactions to build a rigid tubular structure of the rod^26^. In contrast, the D1 domains in FlgE are packed loosely in all directions, allowing the hook to adopt highly curved conformations^23^. FlgG has the GSS in the N-terminal part of domain corresponding to domain Dc of FlgE^25^,^26^. It has been shown that the GSS insertion between Phe-42 and Ala-43 of FlgE in the N-terminal part of domain Dc makes the hook straight, having suggested that the GSS confers the rigidity on the FlgG rod structure^26^. Here, to clarify the effects of the GSS insertion into FlgE on the mechanical property of the hook and motility of *Salmonella* cells, we characterized the *flgE*_+*GSS*_ cells in more detail. We showed that the GSS insertion did not interfere with hook polymerization although the polymerization rate was slower than that of wild-type cells (Fig. 1). The GSS insertion made the hooks straight and rigid (Fig. 3) and hence prevented the flagellar filaments from forming a bundle behind the cell body (Fig. 4), indicating that the GSS insertion made the D1 domains of FlgE tightly packed axially in a way similar to those of FlgG. These results demonstrate that the distinct mechanical properties of the rod and hook, being rigid and flexible, respectively, result from the presence and absence of the GSS in FlgG and FlgE, respectively.

The hook shows polymorphic transformations of its supercoiled form^24^. The curvature and twist of each supercoil have been postulated to depend on the direction of intermolecular D2-D2 interactions^21^. An in-frame deletion variant of FlgE lacking residues 50–59 produces not only normal polyhooks but also nearly straight ones^27^, suggesting that domain Dc of FlgE plays a critical role in polymorphic transformations of the supercoiled form of the hook. Since the GSS insertion into FlgE suppresses the bending flexibility of the hook (Figs 3and 4), it is most likely that the GSS insertion affects the orientations and axial packing interactions of the D1 domain of FlgE. Therefore, we propose that the Dc domain of FlgE acts as a structural switch to coordinate axial packing interactions of the D1 domains with the supercoiling of the hook structure.

## Methods

### Bacterial strains, transductional crosses, DNA manipulations and media

Bacterial strains used in this study are listed in Table 1. P22-mediated transductional crosses were carried out using p22*HTint* as described previously^33^. L-broth (LB) and soft agar plates were prepared as described before^34^,^35^. Ampicillin and tetracycline were added to LB at a final concentration of 100 μg/ml and 15 μg/ml, respectively.

### Motility assay in soft agar

Fresh colonies were inoculated onto soft agar plates and incubated at 30 °C.

### Flagellar protein export assay

*Salmonella cells* were grown at 30 °C with shaking until the cell density had reached an OD_600_ of ca. 1.2–1.6. After centrifugation, the whole cellular and culture supernatant fractions were collected separately. Cell pellets were resuspended in SDS-loading buffer normalized by the cell density to give a constant amount of cells. Proteins in the culture supernatants were precipitated by 10% trichloroacetic acid, suspended in a Tris/SDS loading buffer and heated at 95 °C for 3 min. After SDS-PAGE, immunoblotting with polyclonal anti-FlgD, anti-FlgE, anti-FliK, anti-FlgK, anti-FlgL, or anti-FliC antibody was carried out as described^34^. Detection was done with an ECL Prime immunoblotting detection kit (GE Healthcare).

### Immunostaining of flagellar filaments

The *Salmonella* SJW1103 and MME1001 cells were attached to a cover slip (Matsunami glass, Japan), and unattached cells were washed away with motility medium (10 mM potassium phosphate, 0.1 mM EDTA, and 10 mM sodium lactate; pH 7.0). Flagellar filaments were labeled with polyclonal anti-FliC antibody and anti-rabbit IgG conjugated with Alexa Fluor 594 (Invitrogen) as described previously^36^. After washing twice with the motility medium, cells were observed by an inverted fluorescence microscope (IX-83, Olympus) with a 100× oil immersion objective lens (UPLSAPO 100XO, NA 1.4, Olympus) and an Electron-Multiplying Charge-Coupled Device (EMCCD) camera (iXon Ultra 897, Andor Technology) as described before^37^.

### Visualization of flagellar filaments of swimming cells

The *Salmonella* SJW1103 and MME1001 cells were grown at 30 °C with shaking until the cell density had reached an OD_600_ of ca. 1.6–1.8. The cells were washed twice with motility medium (pH 7.0) and resuspended in 100 μl of motility medium (pH 7.5) containing 50 mM sodium hydrogen carbonate and 1 mg/ml of Cy3 mono-reactive succinimidyl ester dye (GE Healthcare). After mixing at room temperature for 60 min by a rotator with slow rotation at 5 rpm in the dark, stained cells were washed three times with the motility medium (pH 7.0) and observed by an inverted fluorescence microscope (IX-71, Olympus) with a 150× oil immersion objective lens (UAPO 150XOTIRFM, NA1.45, Olympus) and an EMCCD camera (iXon^EM^ + 897-BI, Andor Technology). Cy3 was excited by a 150 mW Ar laser (35-LAL-515, Melles Griot), and emission was detected through a 610/75 nm band-pass filter (Chroma).

### Preparation of hook-basal body structures

Hook-basal bodies and polyhook-basal bodies were prepared as described before^38^. Cells were grown in LB at 37 °C with shaking until the cell density had reached an OD_600_ of ca. 1.0–1,3. The cells were harvested and suspended in ice-cold 0.1 M Tris-HCl, pH 8.0, 0.5 M sucrose. EDTA and lysozyme were added at the final concentrations of 10 mM and 1.0 mg/ml, respectively. The cell suspensions were stirred for 30 min at 4 °C, and then the cell membranes were solubilized on ice for 1 hour by adding Triton X-100 and MgSO_4_ at final concentrations of 1.0% and 10 mM, respectively. The pH of the cell lysates was adjusted to 10.5 with 5 N NaOH. After centrifugation (10,000 g, 20 min, 4 °C), the lysates were ultracentrifuged (45,000 g, 60 min, 4 °C), and the pellets were resuspended in 10 mM Tris-HCl, pH 8.0, 5 mM EDTA, 1% Triton X100. The hook-basal bodies and polyhook-basal bodies were collected from a fraction of 20–50% sucrose density-gradient centrifugation. After ultracentrifugation at 60,000 g for 60 min, the pellet was resuspended in 10 mM Tris-HCl, pH8.0, 5 mM EDTA, 0.1% Triton X100. Samples were negatively stained at room temperature with 2% phosphotungstic acid (pH 6.5) on carbon-coated copper grids. Electron micrographs were recorded with a JEM-1011 transmission electron microscope (JEOL, Tokyo, Japan) operated at 100 kV and equipped with a F415 CCD camera (TVIPS, Gauting, Germany) at a magnification of x5,500, which corresponds to 2.75 nm per pixel.

## Additional Information

**How to cite this article**: Hiraoka, K. D. *et al*. Straight and rigid flagellar hook made by insertion of the FlgG specific sequence into FlgE. *Sci. Rep.* **7**, 46723; doi: 10.1038/srep46723 (2017).

**Publisher's note:** Springer Nature remains neutral with regard to jurisdictional claims in published maps and institutional affiliations.

## Supplementary Material

Supplementary Information

Supplementary Movie S1

Supplementary Movie S2

## Figures and Tables

**Table 1:** Strains and Plasmids used in this study.

*Salmonella* strains	Relevant characteristics	Source or reference
***Salmonella***		
SJW1103	Wild-type for motility and chemotaxis	33
MMEK001	∆*flgE* ∆*fliK*::*tetRA*	39
MME1001	*flgE*::*GSS*	26
MMEK1001	*flgE*::*GSS* ∆*fliK*::*tetRA*	26
NME001	∆*flgE*	39
