# Supplementary material for: Straight and rigid flagellar hook made by insertion of the FlgG specific sequence into FlgE
Source: Sci Rep. 2017 Apr 21;7:46723. doi: 10.1038/srep46723 (PMC5399456; doi:10.1038/srep46723)
Supplement: Supplementary Information [file srep46723-s1.pdf]

Supplementary Information

Straight and rigid flagellar hook made by insertion of the FlgG specific sequence into FlgE

Koichi D. Hiraoka, Yusuke V. Morimoto, Yumi Inoue, Takashi Fujii, Tomoko Miyata, Fumiaki Makino, Tohru Minamino and Keiichi Namba

Movie S1 Free swimming of SJW1103 (WT) stained with a Cy3 mono-reactive dye in liquid media.

Movie S2. Free swimming of MME1001(*flgE*$_{+GSS}$) stained with a Cy3 mono-reactive dye in liquid media.
